# Investigation of temperature conditions in Swiss urban and suburban microhabitats for the overwintering suitability of diapausing *Aedes albopictus* eggs

**DOI:** 10.1186/s13071-018-2803-y

**Published:** 2018-03-27

**Authors:** Damiana Ravasi, Valeria Guidi, Eleonora Flacio, Peter Lüthy, Karl Perron, Samuel Lüdin, Mauro Tonolla

**Affiliations:** 1 Laboratory of Applied Microbiology, University of Applied Sciences and Arts of Southern Switzerland, via Mirasole 22A, 6500 Bellinzona, Switzerland; 2 Institute of Microbiology, ETH Zurich, Vladimir-Prelog-Weg 1-5/10, 8093 Zurich, Switzerland; 3 Microbiology Unit, Plant Biology Department, Sciences III University of Geneva, Quai Ernest-Ansermet 30, 1211 Geneva, Switzerland; 4 Federal Office for Civil Protection, Spiez Laboratory, Biology Division, 3700 Spiez, Switzerland

**Keywords:** *Aedes albopictus*, Microclimate, Temperature, Breeding site, Diapause

## Abstract

**Background:**

In Switzerland, the invasive Asian tiger mosquito, *Aedes albopictus*, is firmly established in the Canton of Ticino, south of the Alps. According to a large-scale distribution model developed in 2013, suitable climatic conditions for the establishment of *Ae. albopictus* north of the Alps are found in Basel and Geneva while Zurich appears to be characterized by winters currently being too cold for survival of diapausing eggs. However, the spatial resolution of large-scale distribution models might not be sufficient to detect particular climatic conditions existing in urban settings, such as the presence of microclimatic temperatures, which may positively influence the probability of diapausing eggs to overwinter. In order to investigate this, microclimatic monitoring of potential diapausing sites (i.e. catch basins) and external controls was performed in January 2017 in Ticino and within the cities of Basel, Geneva and Zurich.

**Results:**

Mean January temperatures in catch basins of Basel, Geneva and Zurich were always higher than the -1 °C temperature threshold previously set for survival probability of diapausing eggs, while mean January temperatures were below -1 °C in several catch basins south of the Alps, where *Ae. albopictus* eggs currently overwinter. The catch basin absolute January daily minimum temperatures both south and north of the Alps were in general higher than the external control temperatures. Absolute January daily minimum temperatures in catch basins in Basel, Geneva and Zurich were always above -10 °C, indicating that diapausing *Ae. albopictus* eggs could potentially survive winter nights in urban areas north of the Alps.

**Conclusions:**

The findings confirmed previous conclusions that urban catch basins can provide favourable conditions for overwintering of diapausing eggs compared to more cold-exposed sites. The results confirmed the presence of suitable winter conditions for the establishment of *Ae. albopictus* in the cities of Basel and Geneva. In addition, the microclimate-scale analysis added new information compared to the previous large-scale prevision model by showing that also the city of Zurich could provide winter conditions suitable for the establishment of *Ae. albopictus*. This illustrates the importance of the resolution of climate data in using models to predict *Ae. albopictus* distribution.

**Electronic supplementary material:**

The online version of this article (10.1186/s13071-018-2803-y) contains supplementary material, which is available to authorized users.

## Background

Since its appearance in Italy in the 1990s, the Asian tiger mosquito, *Aedes albopictus* (Skuse, 1894), has continuously extended its distribution in southern and central Europe [1, 2] causing great concern because of its vectorial capacity for multiple pathogens [3–6]. The expansion of this mosquito species northwards is currently limited by environmental parameters, such as winter and summer temperatures, and precipitation patterns [7, 8]. In particular, low winter temperatures represent a key limiting factor for the survival of diapausing eggs [9–12]. However, due to global warming, *Ae. albopictus* is expected to expand its range further northwards, as predicted by a number of distribution models [9, 12–16].

In Switzerland, *Ae. albopictus* is firmly established since 2007 in the southern part of the country within the main urban and suburban areas [17], where an extensive surveillance and control programme is ongoing [18, 19]. Climatic conditions in Switzerland differ between the southern and northern parts due to the alpine rim acting as a climatic barrier. The climate of southern Switzerland is characterized by milder winters compared to the northern part. A potential establishment of the mosquito in the northern part of the country could therefore be prevented by harsher winter conditions.

In 2013, a large-scale distribution model was developed to identify the most suitable areas for invasion and establishment of *Ae. albopictus* in Switzerland, based on current climatic conditions and expected future scenarios [12]. January mean temperatures were used as a threshold to estimate the survival chances of overwintering diapausing eggs, whereas the annual mean temperature was used as a threshold to estimate population stability. According to the model’s predictions, the region of Geneva and areas surrounding Lake Léman in western Switzerland provide suitable climatic conditions for the establishment of *Ae. albopictus*. In northern Switzerland, parts of the Rhine Valley, including the city of Basel, and of the Swiss Plateau, including the city of Zurich, appear to be suitable for the survival at least for adults of *Ae. albopictus* [12]. However, most of these areas (e.g. the city of Zurich) seem to be characterized by winters currently being too cold for survival of diapausing eggs [12].

Although large-scale distribution models can be useful for risk assessment of *Ae. albopictus* at a regional level, predictions are usually based on temperature data collected either through remote sensing or from existing weather stations [16]. The spatial resolution obtained through these sources might therefore not be sufficient to detect the particular microclimatic conditions existing in urban and suburban settings, where the preferred establishment of *Ae. albopictus* takes place. For instance, suitability analysis in Neteler et al. [12] was based on remotely sensed land surface temperature data with a spatial resolution of 250 m. The complexity of the urban climate, where air temperatures can vary by several degrees within very short distances, may therefore influence the development and life-cycle of *Ae. albopictus*. In fact, temperature characterization within ordinary catch basins, considered the most productive breeding site for *Ae. albopictus* in Italy, showed that temperatures in catch basins in winter were always significantly higher than the external temperature, thus favouring survival of diapausing eggs [20].

Thermal differences among microhabitats and within diapausing sites, such as catch basins, hence could play a fundamental role in the estimation of overwintering and survival rates. The presence of urban heat islands along with milder winter conditions of urban microhabitats may thus increase the probability of diapausing eggs to overwinter and enhance the spread of *Ae. albopictus* to other Swiss cities. The present study aimed to identify the main winter thermal characteristics in proximity to and inside catch basins, in several Swiss towns and cities south and north of the Alps, and to compare the results with previous large-scale estimates of *Ae. albopictus* establishment in Switzerland. A finer-scale and more realistic estimation of the most suitable areas allowing progressive invasion by this species is considered crucial for the establishment of appropriate surveillance and control plans. The study was conducted in January 2017, the month in which the coldest average temperatures of the last 30 years were recorded north of the Alps [21].

## Methods

**Fig. 1:**
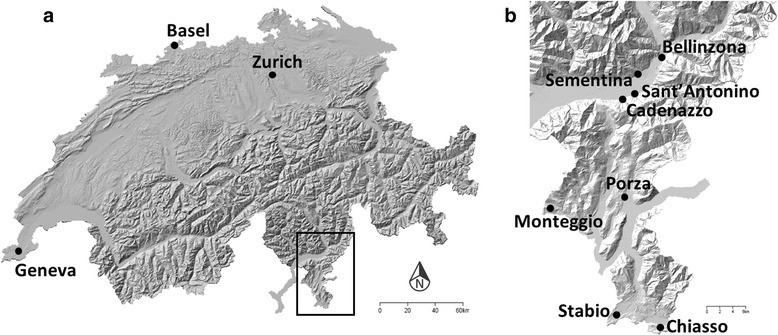
Map of Switzerland with study locations (circles) north (**a**) and south (**b**) of the Alps. Map modified from https://map.geo.admin.ch/

Microclimatic monitoring was performed in eight locations south of the Alps (Canton of Ticino; *Ae. albopictus* established) and in three cities north of the Alps (Basel, Geneva and Zurich; *Ae. albopictus* not established) (Fig. 1). Study sites in Ticino were mostly located in suburban areas (consisting mainly of houses with private garden located in peri-urban area), except for Chiasso, where the monitoring was carried out in an urban context (consisting of apartment blocks and commercial and industrial areas) (Table 1). The study sites were selected based on the presence of established *Ae. albopictus* populations ([19] and Eleonora Flacio, pers. comm.), and on the variability of mean temperatures in January, spanning from colder sites such as Monteggio to warmer sites such as Bellinzona and Sementina (Table 1). In Basel, microclimatic monitoring was performed exclusively in urban area, while in Geneva and Zurich study sites included both urban areas (GEN1, GEN2, GEN4, ZUR1, ZUR2 and ZUR3) and suburban areas (GEN3, GEN5 and ZUR4). Study sites GEN3 and GEN5 were located in private houses in Collonge-Bellerive and Carouge, respectively, while ZUR4 was located on a farm in Unterengstringen, within the agglomeration of Zurich.

**Table 1:** Characteristics of the study sites selected for microclimatic monitoring in Switzerland

Location	Study site	Position^a^	No. of breeding sites	Habitat type	Mean January temperature ± SD (°C)^b^
Bellinzona	BEL	46.198240N, 9.018700E	3	Suburban	2.14 ± 1.59
Cadenazzo	CAD	46.148033N, 8.951583E	3	Suburban	0.34 ± 2.21
Chiasso	CHI	45.834400N, 9.017517E	2	Urban	-0.27 ± 2.06
Monteggio	MON	45.990067N, 8.819633E	3	Suburban	-2.06 ± 2.28
Porza	POR	46.028067N, 8.951117E	2	Suburban	-0.47 ± 2.18
Sant’Antonino	SAN	46.154700N, 8.977533E	3	Suburban	0.52 ± 2.43
Sementina	SEM	46.181517N, 8.996383E	3	Suburban	2.02 ± 1.92
Stabio	STA1	45.852940N, 8.938820E	3	Suburban	-0.09 ± 1.82
	STA2	45.851767N, 8.936550E	3	Suburban	-0.29 ± 1.78
	STA3	45.851817N, 8.939567E	3	Suburban	-0.02 ± 1.73
Basel	BAS	47.561170N, 7.571110E	10	Urban	-0.26 ± 2.67
Geneva	GEN1	46.196800N, 6.135278E	2	Urban	1.75 ± 2.00
	GEN2	46.196667N, 6.135000E	2	Urban	3.39 ± 1.74
	GEN3	46.254444N, 6.198056E	2	Suburban	6.60 ± 0.80
	GEN4	46.189167N, 6.145833E	2	Urban	0.61 ± 2.22
	GEN5	46.182293N, 6.115763E	2	Suburban	0.51 ± 1.85
Zurich	ZUR1	47.392130N, 8.506740E	1	Urban	-1.56 ± 2.99
	ZUR2	47.391870N, 8.509120E	2	Urban	-1.73 ± 2.88
	ZUR3	47.394070N, 8.511830E	2	Urban	-1.29 ± 2.96
	ZUR4	47.415260N, 8.459130E	2	Suburban	-1.64 ± 3.30
Cadenazzo	WS	46.16050N, 8.93260E	–	Suburban	0.84 ± 1.82
Chiasso	WS	45.83333N, 9.01666E	–	Urban	-0.38 ± 2.20
Stabio	WS	45.84338N, 8.93217E	–	Suburban	-0.75 ± 2.34
Basel	WS	47.54104N, 7.58356E	–	Suburban	-1.68 ± 3.42
Geneva	WS	46.24752N, 6.12775E	–	Suburban	-1.15 ± 2.40
Zurich	WS	47.38333N, 8.53333E	–	Urban	-1.43 ± 3.41

^a^Position refers to the GPS coordinates of the external control or the weather station

^b^Based on temperature data of external controls and weather stations recorded in January 2017

*Abbreviations*: *SD* standard deviation, *WS* weather station

For each study site, microclimatic monitoring was performed on one to 10 potential breeding sites and one external control (Table 1). External controls were located in the proximity of monitored breeding sites. Ordinary urban and suburban catch basins were monitored in Ticino, as they represent one of the most favoured breeding sites for *Ae. albopictus* [18, 22]. In Basel, Geneva and Zurich, potential breeding sites included urban catch basins, but also smaller-capacity private catch basins.

Microclimatic temperatures were collected from December 2016 to April 2017 in the selected sites using waterproof data loggers (HOBO® Pendant® Temperature/Light Data Logger, Onset Computer Corporation, Bourne, USA), with acquisition interval set at 30 min. For monitoring of known and potential breeding sites, the sensors were attached to the catch basin’s grid and left hanging inside the basin, above the water level. The external control sensors were placed in proximity of a monitored catch basin, 1–2 m above ground. Temperature loggers were put inside small transparent plastic tubes to protect them from being directly exposed to rain. The tubes were pierced several times in order to allow airflow.

Temperature data collected by field loggers were compared to temperature data of permanent weather stations in six locations south and north of the Alps (i.e. Cadenazzo, Chiasso, Stabio, Basel, Geneva and Zurich). Temperature data of permanent weather stations were collected at 2 m above ground. The weather stations belong to the automatic monitoring network of the Federal Office of Meteorology and Climatology MeteoSwiss, and their geographical position is indicated in Table 1.

The analysis was restricted to data recorded in January 2017, the month with the lowest mean temperatures of the winter 2016–2017 [21]. Multiple catch basins of a study site were treated as replicates and recorded data were pooled for the analysis. Daily temperature range was calculated as the difference between maximum and minimum temperature. The summary statistics and graphs were prepared in Microsoft Excel. Statistical analyses were performed using the software IBM SPSS Statistics for Windows, Version 24.0. The nonparametric Mann-Whitney U-test was used to compare data obtained for catch basins to external controls at each study site. The same test was used to compare catch basin temperature data between Ticino, Basel, Geneva and Zurich and to compare temperature data between catch basins, external controls and permanent weather stations of six locations. Spearman’s rank correlation coefficient was used to express the correlation between temperature data of catch basins and permanent weather stations. A *P* value < 0.05 was determined as significant.

## Results

**Fig. 2:**
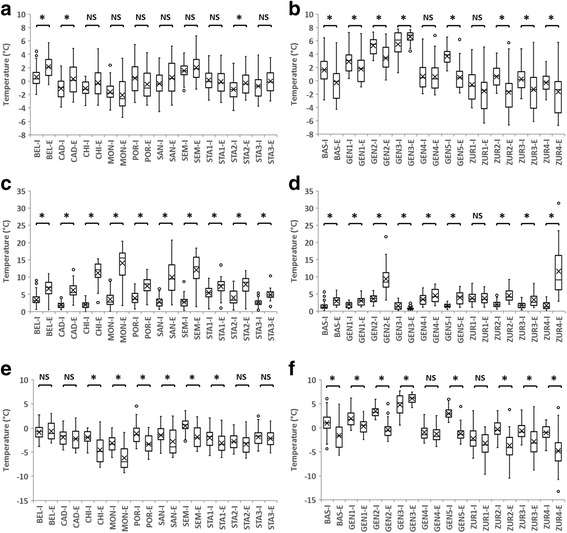
Temperatures recorded by microclimatic data loggers at selected study sites south (**a**, **c** and **e**) and north (**b**, **d** and **f**) of the Alps in January 2017: daily mean temperature (**a** and **b**), daily temperature range (**c** and **d**) and daily minimum temperature (**e** and **f**). The letters I and E following the study site’s name indicate whether the temperature was measured inside catch basins or as external control, respectively. The box plots represent median (solid lines within boxes), mean (x marks within boxes), 25th and 75th percentiles (boxes) and minimum and maximum values (whiskers). *Significant differences between groups. *Abbreviation*: NS, not significant

**Fig. 3:**
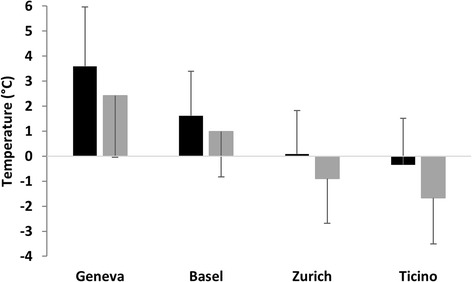
Mean January temperatures (black columns) and average January minimum temperatures (grey columns) in all catch basins south (Ticino) and north (Basel, Geneva and Zurich) of the Alps. Standard deviations are represented by black bars

The analysis was based on temperatures recorded in January 2017 by microclimatic data loggers at selected study sites south (Canton of Ticino) and north of the Alps. Recorded temperature data are presented in Additional file 1: Table S1. Mean January temperatures in Ticino catch basins varied from < -1 °C in Monteggio (-1.63 °C), Stabio (-1.23 °C), Cadenazzo (-1.12 °C) and Chiasso (-1.09 °C), to 1.55 °C in Sementina. Mean January temperatures of external controls ranged from -2.06 °C in Monteggio to > 2 °C in Sementina (2.02 °C) and Bellinzona (2.14 °C) (Fig. 2a). Mean January temperatures in catch basins north of the Alps varied from -0.58 °C in Zurich (study site ZUR1) to >5 °C in Geneva (GEN2, 5.28 °C; GEN3 5.48 °C). Mean January temperatures of external controls ranged from < -1.5 °C in Zurich (ZUR2, -1.73 °C; ZUR4, -1.64 °C; ZUR1, -1.56 °C) to > 6 °C in Geneva (GEN3 6.60 °C) (Fig. 2b). Overall, mean January temperatures in catch basins differed significantly (*P* < 0.05 in all two-sample tests) between Ticino, Basel, Geneva and Zurich. The warmest mean January temperatures were recorded in Geneva catch basins, followed by Basel and Zurich (Fig. 3). Mean January temperatures appeared to be lower in Ticino compared to the study sites north of the Alps (Fig. 3).

For each study site, we compared the daily mean January temperatures of catch basins with the external control. In Ticino, daily mean temperatures in catch basins were higher than external controls in three study sites (Monteggio, Porza and Stabio1), although not significantly so. In the other seven sites, daily mean temperatures in catch basins were lower than in external controls, significantly so in three cases (i.e. Bellinzona, Cadenazzo and Stabio2) (Fig. 2a). The lower daily mean temperatures in catch basins compared to external controls can be explained by the fact that daily temperature range was significantly smaller in catch basins compared to external controls (Fig. 2c). Indeed, larger variation between maximum and minimum daily temperatures will result in higher daily mean temperature, and *vice versa*. In study sites north of the Alps, daily mean January temperatures in catch basins were mostly significantly higher than external controls, except in GEN3 where they were significantly lower (Fig. 2b). The daily temperature range was significantly smaller in catch basins than external controls, except for sites ZUR1 (not significantly higher) and GEN3 (significantly higher). The largest daily temperature range was recorded at the external control of suburban study site ZUR4 (Fig. 2d).

Absolute January minimum temperatures in Ticino catch basins ranged from -6.12 °C in Monteggio to -2.73 °C in Sementina (Fig. 2e). Absolute minimum temperatures of external controls ranged from -9.25 °C in Monteggio and -8.18 °C in Chiasso to -3.08 °C in Bellinzona. At each study site, absolute daily minimum temperatures were always higher in catch basins compared to external controls, except for Bellinzona, and in most cases significantly so (Fig. 2e). Absolute January minimum temperatures in catch basins north of the Alps varied from -6.37 °C in ZUR1 urban study site to 1.66 °C in GEN2 urban site. At external controls, absolute January minimum temperatures varied from -13.26 °C in ZUR4 suburban site and -10.49 °C in ZUR2 urban site to 4.21 °C in GEN3 suburban site. For each study site, absolute daily minimum temperatures in catch basins were always higher than in the external controls, except for study site GEN3, and mostly significantly so (Fig. 2f). Overall, average January minimum temperatures in catch basins differed significantly (*P* < 0.05 in all two-sample tests) between Ticino, Basel, Geneva and Zurich. The warmest average January minimum temperatures were recorded in Geneva catch basins, followed by Basel and Zurich (Fig. 3). Average January minimum temperatures appeared to be lower in Ticino compared to the study sites north of the Alps (Fig. 3).

Mean January temperatures and average January minimum temperatures of catch basins and external controls in six locations were compared to permanent weather stations (Table 2). Mean January temperatures of catch basins were significantly higher than the corresponding weather stations in three locations (Basel, Geneva and Zurich) while they were significantly lower in one location (Cadenazzo). Mean January temperatures of external controls did not show significant difference from the weather stations, except for Geneva (external control temperature significantly higher than weather station). The correlation between mean January temperatures of catch basins and weather stations was high and always significant (Table 2). Average January minimum temperatures of catch basins were always significantly higher than the corresponding weather station. Average January minimum temperatures of external controls were significantly higher than the corresponding weather stations in Cadenazzo, Stabio, Basel and Geneva. The correlation between average January minimum temperatures of catch basins and weather stations was high (except for Geneva) and always significant (Table 2).

**Table 2:** Comparisons of mean January temperatures and average January minimum temperatures between catch basins, external controls and weather stations of six locations south and north of the Alps

Location	Catch basins	External controls	Weather station	Catch basins *vs* weather station	External controls *vs* weather station	Catch basins *vs* weather station
				Mann-Whitney U	Mann-Whitney U	Spearman’s correlation
Mean January temperatures ± SD (°C)^a^						
Cadenazzo	-1.12 ± 1.46	0.34 ± 2.21	0.84 ± 1.82	569.5*	409.0	0.685*
Chiasso	-1.09 ± 1.03	-0.27 ± 2.10	-0.38 ± 2.20	708.5	421.0	0.846*
Stabio	-0.61 ± 1.75	-0.13 ± 1.76	-0.75 ± 2.34	4155.5	1213.0	0.853*
Basel	1.62 ± 1.77	-0.26 ± 2.67	-1.68 ± 3.42	1847.0*	342.0	0.896*
Geneva	3.60 ± 2.37	2.57 ± 2.88	-1.15 ± 2.40	859.0*	753.0*	0.925*
Zurich	0.09 ± 1.74	-1.55 ± 3.01	-1.43 ± 3.41	2259.5*	1915.0	0.856*
Mean January minimum temperatures ± SD (°C)^a^						
Cadenazzo	-1.92 ± 1.39	-2.24 ± 1.93	-3.56 ± 2.09	728.0*	287.0*	0.760*
Chiasso	-1.94 ± 1.28	-4.54 ± 2.41	-5.59 ± 2.36	179.0*	322.0	0.837*
Stabio	-2.21 ± 1.70	-2.88 ± 1.84	-6.02 ± 2.73	1062.0*	468.0*	0.897*
Basel	0.98 ± 1.81	-1.65 ± 2.68	-4.47 ± 3.62	981.0*	241.0*	0.866*
Geneva	2.42 ± 2.46	0.71 ± 3.19	-3.95 ± 2.07	300.0*	322.0*	0.610*
Zurich	-0.89 ± 1.79	-3.66 ± 3.15	-3.42 ± 3.28	1461.0*	1853.0	0.899*

^a^Based on temperature data recorded in January 2017

*Significant Mann-Whitney U outcomes and significant Spearman’s rank correlations coefficients

*Abbreviation*: *SD* standard deviation

## Discussion

The present study aimed to understand whether urban microclimatic conditions within Swiss cities north of the Alps could be suitable for overwintering of *Ae. albopictus* diapausing eggs, thus possibly favouring the establishment of the mosquito. In January 2017, microclimatic monitoring was performed in known *Ae. albopictus* overwintering sites south of the Alps, where *Ae. albopictus* is firmly established, and potential overwintering sites north of the Alps, where the mosquito is currently not present. The microclimate-scale findings were compared to previous large-scale estimates of *Ae. albopictus* establishment in Switzerland [12].

A range of January mean temperature values have been reported as thresholds for the overwintering of *Ae. albopictus* eggs. A January mean temperature of > 0 °C has been generally accepted as delineating overwintering populations for modelling climatic suitability for *Ae. albopictus* [8, 9, 14, 23]. Waldock et al. [23] suggested that in Europe, a cut-off of -4 °C may be more accurate. In Neteler et al. [12] large-scale prevision model for establishment of *Ae. albopictus* in Switzerland, the mean January temperature threshold for survival probability of diapausing eggs was set to 1 °C with a margin of 2 °C. Totally unsuitable conditions were therefore defined as < -1 °C, and totally suitable conditions as > 3 °C. According to the model, suitable conditions for the overwintering of *Ae. albopictus* eggs are present in Basel and Geneva, while in the canton of Zurich the climatic conditions appear currently unsuitable for egg survival [12]. In the present study, mean January temperatures in catch basins of Basel, Geneva and Zurich never dropped below -1 °C, and only in two study sites (i.e. urban site Zurich1 and suburban site Zurich4) they were below 0 °C. Interestingly, mean January temperatures were below -1 °C in several locations, both suburban and urban, south of the Alps (i.e. Monteggio, Stabio, Cadenazzo and Chiasso), where *Ae. albopictus* is already established. Therefore, according to the mean January temperatures recorded in the present study, suitable conditions for overwintering of *Ae. albopictus* diapausing eggs are present not only in the cities of Basel and Geneva, as predicted by Neteler et al. [12], but also in Zurich. These results are even more remarkable considering that mean January temperatures recorded north of the Alps in 2017 were the coldest in the last 30 years [21], which makes the analysis conservative with respect to probability of establishment. This also clearly demonstrates the important impact of thermal islands within big cities.

Absolute minimum temperature is also a major determinant of overwintering mortality in *Ae. albopictus* eggs [20, 24]. Experiments on egg mortality in response to cold temperatures have demonstrated remarkable cold-resistance. A laboratory study by Thomas et al. [11] showed that minimum survival temperatures for temperate *Ae. albopictus* diapausing eggs were -10 °C for long-term exposure (12–24 h) and -12 °C for short-term exposure (1 h), confirming that diapausing *Ae. albopictus* eggs are, or became, cold adapted and are able to survive winter nights down to -10 °C (although only around 10% of eggs hatched after exposure to this temperature). In the present study, the catch basin absolute January daily minimum temperatures both south and north of the Alps were in general significantly higher than the external control temperatures, except for four locations in Ticino (Bellinzona, Cadenazzo, Stabio2 and Stabio3) and three locations north of the Alps (Geneva3, Geneva4 and Zurich1). Furthermore, absolute daily minimum temperatures in catch basins during January both south and north of the Alps never dropped below -7 °C, while they did drop below -10 °C in some external controls (i.e. Zurich2, Zurich4) for one to two consecutive nights, and below -7 °C in two other external controls (i.e. Zurich1, Zurich3) for one night. However, absolute daily minimum temperatures also dropped below -7 °C for several nights in a row in external controls in Ticino locations (i.e. Monteggio and Chiasso) where *Ae. albopictus* eggs do currently overwinter.

The findings of the present study confirmed previous conclusions [20] that winter temperatures in catch basins are higher and more stable compared to external temperatures. Therefore, catch basins represent not only known *Ae. albopictus* breeding sites, but they can also provide a shelter for overwintering of diapausing eggs compared to more cold-exposed sites. In addition, the study showed that minimum temperatures of catch basins were higher compared to nearby permanent weather stations, while mean temperatures did not always show the same pattern. Data recorded by external controls were more similar to data recorded by weather stations; mean temperatures of external controls did not differ significantly from weather stations, although minimum temperatures of external controls were significantly higher in external controls in several locations. Interestingly, there was a strong correlation between mean and minimum temperatures of catch basins and weather stations, suggesting a significant relationship between microclimatic temperatures and temperatures recorded by permanent weather stations, as previously shown by Vallorani et al. [20].

Our results support the prevision made by Neteler et al. [12], based on large-scale distribution model, by confirming the presence of suitable winter conditions for establishment of *Ae. albopictus* in the cities of Basel and Geneva. In addition, our microclimate-scale analysis added new information compared to the large-scale prevision model by showing that the city of Zurich could also provide suitable overwintering conditions for the establishment of *Ae. albopictus*. This illustrates the importance of the resolution of climate data in using models to predict *Ae. albopictus* distribution [23]. As shown in this study, large-scale prevision models of climatic suitability for *Ae. albopictus* do not take into account conditions of urban microhabitats that can provide unforeseen conditions for the survival of eggs of *Ae. albopictus*.

## Conclusions

This study expands the information on suitability of northern Switzerland for the invasion of *Ae. albopictus* by comparing microclimate conditions in known overwintering sites in the Ticino with those still free from *Ae. albopictus* in cities north of the alps. In addition to winter temperature, other environmental parameters such as annual precipitation, mean annual temperature and summer temperature should be studied and integrated to obtain a complete picture of the risk that *Ae. albopictus* gains a foothold north of the Swiss Alps. Temperature thresholds for the survival of overwintering eggs should also be examined in more detail by field studies, observing survival under field conditions during a whole winter period in comparison with short exposure to cold temperatures in the laboratory. Additional ecological parameters to be investigated in relation to climatic conditions in catch basins are the duration of larval developmental stages and mortality rates. Eggs of invasive mosquitoes can be passively spread over long distances through the international trade, while adult individuals can be displaced through road transport. Adults and/or eggs of *Ae. albopictus* have been reported during the warm season from the cantons of Basel and Zurich [25]. *Aedes albopictus* reaches the north of the Alps using the south-north trans-border European traffic, in our case the A2 motorway [25]. A stable population of tiger mosquito has been reported from Freiburg im Breisgau (Germany) since 2015 [26] from where adults could reach Basel through the A5 highway. *Aedes albopictus* specimens have not been reported yet from the canton of Geneva. However, the areas around Lake Geneva could be colonized in the near future through road transport from the French Rhone valley. The invasive pressure combined with the previous and present findings suggest that *Ae. albopictus* will very likely colonize urban areas north of the Alps during the coming years. Although infected mosquitoes have not yet been observed in Switzerland [27], outbreaks of mosquito-borne diseases such as chikungunya and dengue occurred in the neighbouring countries Italy and France [3–5, 28]. It is therefore essential to integrate the impact of microclimates into the existing models in order to obtain more precise predictions on the temporal-spatial dynamics of *Ae. albopictus* along with the transmission potential of arboviruses. Such information is needed to complete monitoring and control tools and risk-based surveillance of *Ae. albopictus* populations.

## Additional file

Additional file 1: **Table S1.** Recorded temperature series for January 2017. Details and names of study sites are reported in Table 1 and Fig. 2. (XLSX 616 kb)

## References

[1] European Centre for Disease Prevention and Control. *Aedes albopictus* - current known distribution in Europe, September 2017. https://ecdc.europa.eu/en/publications-data/aedes-albopictus-current-known-distribution-september-2017. Accessed 20 Nov 2017.

[2] Medlock JM, Hansford KM, Schaffner F, Versteirt V, Hendrickx G, Zeller H (2012). A review of the invasive mosquitoes in Europe: ecology, public health risks, and control options. Vector-Borne Zoonotic Dis..

[3] Angelini R, Finarelli AC, Angelini P, Po C, Petropulacos K, Macini P, et al. An outbreak of chikungunya fever in the province of Ravenna, Italy. Euro Surveill. 2007;12(36). 10.2807/esw.12.36.03260-en. 10.2807/esw.12.36.03260-en 17900424

[4] Gould EA, Gallian P, De Lamballerie X, Charrel RN (2010). First cases of autochthonous dengue fever and chikungunya fever in France: from bad dream to reality!. Clin Microbiol Infect..

[5] Delisle E, Rousseau C, Broche B, Leparc-Goffart I, L’Ambert G, Cochet A, et al. Chikungunya outbreak in Montpellier, France, September to October 2014. Euro Surveill. 2015;20(17). 10.2807/1560-7917.ES2015.20.17.21108. 10.2807/1560-7917.es2015.20.17.21108 25955774

[6] Manica M, Guzzetta G, Poletti P, Filipponi F, Solimini A, Caputo B, et al. Transmission dynamics of the ongoing chikungunya outbreak in central Italy: from coastal areas to the metropolitan city of Rome, summer 2017. Euro Surveill. 2017;22(44). 10.2807/1560-7917.ES.2017.22.44.17-00685. 10.2807/1560-7917.ES.2017.22.44.17-00685 PMC5710132 29113629

[7] Mogi M, Armbruster P, Tuno N, Campos R, Eritja R (2015). Simple indices provide insight to climate attributes delineating the geographic range of *Aedes albopictus* (Diptera: Culicidae) prior to worldwide invasion. J Med Entomol..

[8] Cunze S, Kochmann J, Koch LK, Klimpel S (2016). *Aedes albopictus* and its environmental limits in Europe. PLoS One..

[9] Medlock JM, Avenell D, Barrass I, Leach S (2006). Analysis of the potential for survival and seasonal activity of *Aedes albopictus* (Diptera: Culicidae) in the United Kingdom. J Vector Ecol..

[10] Delatte H, Gimonneau G, Triboire A, Fontenille D (2009). Influence of temperature on immature development, survival, longevity, fecundity, and gonotrophic cycles of *Aedes albopictus*, vector of chikungunya and dengue in the Indian Ocean. J Med Entomol..

[11] Thomas S, Obermayr U, Fischer D, Kreyling J, Beierkuhnlein C (2012). Low-temperature threshold for egg survival of a post-diapause and non-diapause European aedine strain, *Aedes albopictus* (Diptera: Culicidae). Parasit Vectors..

[12] Neteler M, Metz M, Rocchini D, Rizzoli A, Flacio E, Engeler L (2013). Is Switzerland suitable for the invasion of *Aedes albopictus*?. PLoS One..

[13] European Centre for Disease Prevention and Control (ECDC) (2009). Development of *Aedes albopictus* risk maps.

[14] Caminade C, Medlock JM, Ducheyne E, McIntyre KM, Leach S, Baylis M (2012). Suitability of European climate for the Asian tiger mosquito *Aedes albopictus*: recent trends and future scenarios. J R Soc Interface..

[15] Erickson RA, Hayhoe K, Presley SM, Allen LJS, Long KR, Cox SB (2012). Potential impacts of climate change on the ecology of dengue and its mosquito vector the Asian tiger mosquito (*Aedes albopictus*). Environ Res Lett..

[16] Fischer D, Thomas SM, Neteler M, Tjaden NB, Beierkuhnlein C. Climatic suitability of *Aedes albopictus* in Europe referring to climate change projections: comparison of mechanistic and correlative niche modelling approaches. Euro Surveill. 2014;19(6). 10.2807/1560-7917.ES2014.19.6.20696. 10.2807/1560-7917.es2014.19.6.20696 24556349

[17] Wymann MN, Flacio E, Radczuweit S, Patocchi N, Lüthy P. Asian tiger mosquito (*Aedes albopictus*) - a threat for Switzerland? Euro Surveill. 2008;13(10). 10.2807/ese.13.10.08058-en. 10.2807/ese.13.10.08058-en 18445441

[18] Flacio E, Engeler L, Tonolla M, Lüthy P, Patocchi N (2015). Strategies of a thirteen year surveillance programme on *Aedes albopictus* (*Stegomyia albopicta*) in southern Switzerland. Parasit Vectors..

[19] Flacio E, Engeler L, Tonolla M, Müller P (2016). Spread and establishment of *Aedes albopictus* in southern Switzerland between 2003 and 2014: an analysis of oviposition data and weather conditions. Parasit Vectors..

[20] Vallorani R, Angelini P, Bellini R, Carrieri M, Crisci A, Zeo SM (2015). Temperature characterization of different urban microhabitats of *Aedes albopictus* (Diptera Culicidae) in Central-Northern Italy. Environ Entomol..

[21] MétéoSuisse. Bulletin climatologique janvier 2017. Genève; 2017.

[22] Guidi V, Luthy P, Tonolla M (2013). Comparison between diflubenzuron and a *Bacillus thuringiensis israelensis*- and *Lysinibacillus sphaericus*-based formulation for the control of mosquito larvae in urban catch basins in Switzerland. J Am Mosq Control Assoc..

[23] Waldock J, Chandra NL, Lelieveld J, Proestos Y, Michael E, Christophides G (2013). The role of environmental variables on *Aedes albopictus* biology and chikungunya epidemiology. Pathog Glob Health..

[24] Hanson SM, Craig GB (1994). Cold acclimation, diapause, and geographic origin affect cold hardiness in eggs of *Aedes albopictus* (Diptera: Culicidae). J Med Entomol..

[25] Engeler L, Suter T, Flacio E, Tonolla M, Müller P (2017). Koordination der Überwachung und Bekämpfung der Asiatischen Tigermücke und anderer invasiver gebietsfremder Mücken in der Schweiz. Eine Orientierungshilfe mit Empfehlungen zuhanden des BAFU sowie der kantonalen und anderen betroffener Behörden.

[26] Pluskota B, Jöst A, Augsten X, Stelzner L, Ferstl I, Becker N (2016). Successful overwintering of *Aedes albopictus* in Germany. Parasitol Res..

[27] Engler O, Savini G, Papa A, Figuerola J, Groschup MH, Kampen H (2013). European surveillance for West Nile virus in mosquito populations. Int J Environ Res Public Health..

[28] Venturi G, Di Luca M, Fortuna C, Remoli ME, Riccardo F, Severini F, et al. Detection of a chikungunya outbreak in central Italy, August to September 2017. Euro Surveill. 2017;22(39). 10.2807/1560-7917.ES.2017.22.39.17-00646. 10.2807/1560-7917.ES.2017.22.39.17-00646 PMC5709953 29019306

